# Harnessing *Bacillus amyloliquefaciens* for Amazake Production: Comparison with *Aspergillus oryzae* Amazake for Metabolomic Characteristics, Microbial Diversity, and Sensory Profile

**DOI:** 10.3390/foods13132012

**Published:** 2024-06-26

**Authors:** Alejandra Touceda-Suárez, María Touceda-Suárez, Juan-Carlos Arboleya, Pia M. Sörensen

**Affiliations:** 1 Harvard John A. Paulson School of Engineering and Applied Sciences, Harvard University, Cambridge, MA 02138, USA; 2 Basque Culinary Center, Faculty of Gastronomic Sciences, Mondragon University, 20009 Donostia-San Sebastián, Spain; jcarboleya@bculinary.com; 3 Department of Environmental Science, University of Arizona, Tucson, AZ 85721, USA; mtoucedasuarez@arizona.edu; 4 BCC Innovation, Technology Center in Gastronomy, Basque Culinary Center, 20009 Donostia-San Sebastián, Spain

**Keywords:** amazake, fermentation, *Aspergillus oryzae*, *Bacillus amyloliquefaciens*, enzymatic activity, reducing sugars, metabolomics, metagenomics, sensory analysis

## Abstract

Amazake is a traditional, sweet, non-alcoholic Japanese beverage typically produced through koji fermentation by the fungus *Aspergillus oryzae*. However, alternative microorganisms such as *Bacillus amyloliquefaciens* offer potential advantages and novel possibilities for producing similar fermented beverages. This study aimed to replicate the ancestral beverage of amazake by replacing *A. oryzae* (W-20) with *B. amyloliquefaciens* (NCIMB 12077) and comparing their fermentation processes and resulting products. Our results show that the production of amazake with *B. amyloliquefaciens* (ABA) is not only possible but also results in a beverage that is otherwise distinct from traditional amazake (AAO). Saccharification was achievable in ABA at higher temperatures than in AAO, albeit with lower reducing sugar and enzymatic activity values. Amino acids and organic acids were more abundant in AAO, with cysteine being uniquely present in AAO and shikimic acid only being present in ABA. The volatile aroma compound profiles differed between the two beverages, with AAO exhibiting a greater abundance of aldehydes, and ABA a greater abundance of ketones and alcohols. Interestingly, despite these compositional differences, the two beverages showed similar consumer panel acceptance rates. An analysis of their microbial communities revealed pronounced differences between the amazakes, as well as temporal changes in ABA but not in AAO. This study provides promising insights into harnessing the potential of *B. amyloliquefaciens* as the primary microorganism in the fermentation process of amazake-like beverages, marking an important advancement in the field of fermented low-alcohol beverage production, with possible applications in other fermented foods.

## 1. Introduction

Food fermentation has a long history as a method for producing food with a longer shelf life, enhanced taste, and improved nutritional value. In Japan, a variety of traditional fermented foods are produced using koji. Koji is derived from the fermentation of cereals or legumes—most commonly rice, wheat, or soybeans—by a single microorganism culture of the filamentous fungus *Aspergillus oryzae*, also known as koji mold [1]. The koji mold is rich in secreted enzymes that can be applied in downstream processes. Depending on the substrate and the fermentation process, it can yield a range of culturally significant food products such as miso, sake, and amazake.

Amazake, a traditional Japanese sweet, non-alcoholic drink, has been consumed for over a thousand years [2]. It is produced by adding water and steamed rice to rice koji and incubating at 55–60 °C in an anaerobic environment for several hours [3]. The primary process in amazake production is saccharification, in which rice starches are transformed into simple sugars by enzymes released during the koji fermentation [4]. Amazake’s rising popularity in recent years can be attributed in part to its reported health benefits, such as its antifatigue effect and skin barrier functions, among others [5,6,7]. Despite its long history and popularity, many aspects of traditional amazake production remain unknown. For example, the microbial diversity and protease activity are unexplored, and the amylase activity has only been reported on during the early stages of the fermentation process [4]. This study aims to investigate the dynamics of the fermentation at a deeper level.

*A. oryzae* and other molds are highly valued for their ability to secrete enzymes and are, thus, often considered the default option for processes where exogenous enzymatic effects are desired. In contrast, although bacteria are common in many kinds of food fermentations, they are often underutilized for their ability to secrete enzymes [8]. However, bacterial enzymes can be produced under a wide range of conditions. By exploring alternative microorganisms adaptable to diverse environments, we aim to expand the range of microbial candidates for fermentation processes [9,10]. Through investigating such alternatives [9], there is potential not only to improve the efficiency and scalability but also to enhance the gastronomical and nutritional benefits in amazake manufacturing.

One such microorganism is *Bacillus amyloliquefaciens*, an endospore-forming bacterium commonly found in soil, water, and various food products [11]. Apart from its antifungal function, *B. amyloliquefaciens* has been shown to suppress toxic substances produced by other microorganisms such as *Bacillus cereus* [12]. *B. amyloliquefaciens* is known in the food processing sector for its ability to hydrolyze compounds and synthesize various enzymes (including thermoresistant enzymes), proteins, and carbohydrates, as well as for its prebiotic and probiotic abilities [13]. Although *B. amyloliquefaciens* is known in the food industry, its use as a primary organism in fermentation is relatively unexplored. Moreover, the thermoresistant enzymes synthesized by *B. amyloliquefaciens* [14] offer potential benefits in fermented food production, where survival and functionality at higher temperatures are common challenges [15,16].

Furthermore, while the primary fermentation microbe plays a major role in shaping the characteristics of a fermented food, other microorganisms present in the culture can also impact the final product [9,10]. For example, although traditional amazake is primarily produced with *A. oryzae*, this microbe is part of a larger microbial community that has been reported on in koji [17] but remains unknown in amazake. Thus, it can be expected that fermented foods produced with other microorganisms such as *B. amyloliquefaciens* would also support distinct microbial communities; if so, what are they and how might they contribute to the final product? Understanding the contributions of these secondary microorganisms could provide further insights into the fermentation process and the quality of the final product.

In this study, we investigated the production of amazake using *A. oryzae* (W-20) and *B. amyloliquefaciens* (NCIMB 12077) strains under laboratory conditions to address several questions. (i) Can *B. amyloliquefaciens* effectively ferment rice to produce amazake? (ii) If so, how does the fermentation processes compare between the two microorganisms? (iii) Are the sensory properties and compositional properties such as the sugar content, enzyme activity, and metabolomic profile of amazake produced by *B. amyloliquefaciens* comparable to those of *A. oryzae*? (iv) Are the microbial communities in amazake produced by *B. amyloliquefaciens* and *A. oryzae* different, and if so, how?

## 2. Materials and Methods

### 2.1. Overall Methodology

This study was originally developed by the authors for the first author’s master’s thesis [18], which covered the same topic as this paper. Discussions with the authors about the content in the thesis and this study have since inspired a similar amazake investigation as part of a related study [19].

### 2.2. Chemicals and Reagents

All chemicals outlined in this protocol were of analytical grade and purchased from Sigma-Aldrich (St. Louis, MO, USA), unless otherwise stated.

### 2.3. Microorganisms and Preparation of Inoculum

The *A. oryzae* (W-20), in powdered form, was obtained from Higuchi Matsunosuke Shoten (Abeno, Osaka, Japan). The *B. amyloliquefaciens* (NCIMB 12077) was obtained from the National Collection of Industrial Food and Marine Bacteria (NCIMB Ltd., Aberdeen, UK).

The *A. oryzae* culture did not require pretreatment before inoculating the rice, and the spores were used for the inoculum. The *B. amyloliquefaciens* was grown on Luria–Bertani (LB) agar plates at 37 °C for 24 h [20]. LB broth (10 mL) was inoculated with a 24 h old colony and grown for 24 h at 37 °C with a shaking speed of 110 RPM [14]. The culture was fed after 24 h with 100 mL LB broth and kept for 8 h at the same conditions before harvesting.

### 2.4. Amazake Production

The amazake samples were prepared as follows. First, the kojis were prepared by soaking rice (300 g, short-grain; Koshihikari, Wismettac Asian Foods, Inc., Santa Fe Springs, CA, USA) in filtered tap water (1.2 L) for 12 h at 4 °C and then drained. Next, the rice was submerged in filtered tap water several times until the water had a clear color (with modifications from Gil et al. (2018) [21]). The rice was then steamed with a bamboo steamer (Reishunger, Inc., Bremen, Germany) (100 °C, 90 min) and separated by hand under sterile conditions while still warm to avoid clumping. After reaching 30 °C, the rice was divided in two and inoculated with the fungal strain *A. oryzae* (0.1%, *w*/*w*) and the *B. amyloliquefaciens* inoculum (2.0%, *v*/*w*); the initial viable cell concentration was not noted [22]. The koji samples were incubated at 35 °C and 98% humidity for 36 h [23].

Amazake was prepared by combining steamed rice (300 g, prepared as above) with either of the two kojis (600 g) and filtered tap water (300 g), followed by saccharification in a constant temperature bath at 55 °C or 70 °C [3] for 0, 8, 16 and 24 h. The temperatures were chosen to reflect traditional amazake recipes (55 °C) and the impact of the thermoresistant amylases of the *B. amyloliquefaciens* (70 °C) [14]. After collection, the samples were blended (Breville Control Grip Immersion Blender) and stored at –19 °C until their analysis. All experiments were performed in triplicate.

### 2.5. pH Measurements

The pH measurements were conducted using a pH meter (Mettler Toledo, Model EL20, Columbus, OH, USA).

### 2.6. Extraction and Quantification of Reducing Sugars by Spectrophotometry

The reducing sugar (RS) content was assayed following the method described by Burgos Montañez [24], with modifications in the extraction method.

Each amazake sample (1%, *w*/*v*) was extracted with distilled water by shaking at 120 RPM for 10 min at room temperature. The mixture was then centrifuged at 5000 RPM for 5 min at 23 °C, and the supernatants were filtered using 0.2 µm Acrodisc^®^ syringe filters (Pall Corporation, Port Washington, NY, USA). The filtered supernatants (0.25 mL) and the 3,5-dinitrosalicylic acid reagent (DNS) (0.25 mL) were combined and boiled at 100 °C for 5 min. The reaction was stopped immediately by cooling in an ice bath to room temperature and then mixed with 2.5 mL of distilled water. The absorbance was read at 540 nm using a Pharmacia Biotech Ultrospec 2000 UV–visible spectrophotometer. The standard curve and results are expressed in terms of the RS content (g/L).

### 2.7. Extraction and Quantification of Enzyme Activity by Spectrophotometry

Enzyme activity assays were conducted for α-amylase and protease. The amazake samples were extracted following the method described by Da et al. [22]. Each amazake sample (10 g) was extracted with 90 mL of distilled water by shaking at 120 RPM and 30 °C for 60 min. Next, the mixture was centrifuged at 5000 RPM and 4 °C for 5 min and the supernatants filtered using 0.2 µm Acrodisc^®^ syringe filters.

#### 2.7.1. Protease Activity

The protease activity was assayed following the method described by Gil et al. [21], with some modifications to the incubation time of the first reaction. The assay was conducted as follows. The supernatants of the amazake extract (1 mL) and a 0.65% *w*/*v* casein solution (5 mL) were mixed and incubated at 37 °C for 20 min. The reaction was then stopped with 0.4 M trichloroacetic acid (5 mL), and the samples were incubated at 37 °C for 30 min. Next, the samples were centrifuged at 1100 RPM for 5 min and then filtered using 0.2 µm Acrodisc^®^ syringe filters. The filtrate (2 mL), Folin’s solution (1 mL), and 0.4 M of sodium carbonate (5 mL) were mixed and incubated at 30 °C for 30 min. After the incubation period, the absorbance of the reaction mixture was observed at 660 nm using a Pharmacia Biotech Ultrospec 2000 UV–visible spectrophotometer. One unit of protease activity was defined as the amount of protease required to release 1 µg of tyrosine per minute from 0.65% *w*/*v* of casein.

#### 2.7.2. α-Amylase Activity

The ɑ-amylase activity of the amazake was determined using DNS as described by Beckman et al. [25] with modifications; the temperature and time of the enzyme reaction were changed (10 min at 55 °C for 3 min at 20 °C). The supernatants were 10-fold serially diluted in distilled water, and 1 mL of each diluted supernatant was incubated with 1 mL of 1% soluble starch solution in 20 mM of sodium phosphate buffer with 6.7 mM sodium chloride (pH 6.9) for 3 min at 20 °C, with agitation at 80 RPM. Then, 1 mL of 96 mM DNS was added and the samples were boiled at 100 °C for 15 min. The reaction was stopped immediately via cooling on ice to room temperature and then mixed with 9 mL of distilled water. The absorbance was recorded at 540 nm using a Pharmacia Biotech Ultrospec 2000 UV–visible spectrophotometer. One unit of ɑ-amylase was defined as the quantity of ɑ-amylase that induced a change of 1 mg of maltose in a 1% soluble starch solution in 1 min [22].

### 2.8. Primary Metabolite Profiling with Liquid Chromatography–Mass Spectrometry

A liquid chromatography–mass spectrometry (LC-MS/MS) analysis was performed on amazakes made with *A. oryzae* and *B. amyloliquefaciens* kojis fermented at 55 °C for 16 h. All samples were analyzed in triplicate. Steamed white rice was used as a negative control. All solvents used were of LCMS grade from Sigma-Aldrich.

The extraction of the samples for the LC-MS/MS analysis followed the protocol outlined by Da et al. (2016) [22], with modifications to the extraction method and duration. The amazake (100 mg) samples were transferred to Eppendorf tubes and 1 mL of 80% methanol was added to each sample. The samples were homogenized for 20 min in an ultrasound bath, incubated at −20 °C for 2 h, and then centrifuged at 1100 RPM for 10 min [22]. The supernatants were transferred to new Eppendorf tubes and dried under a N_2_ flow. The samples were resuspended in 100 µL of 25% acetonitrile in water. After centrifugation, the supernatants were transferred to AQ glass micro-inserts. A mix of pure analytical standards was also prepared to confirm the identification of the targets.

The extracts were analyzed via LC-MS/MS (Thermo Qexactive Plus/Ultimate 3000) using a Hilicon iHILIC 150 × 2.1 mm column. The mobile phase consisted of 20 mM of ammonium carbonate, 0.1% ammonium hydroxide in water (solution A), and 97% acetonitrile in water (solution B). A linear gradient elution program was used with a dry flow rate of 0.15 mL/min as follows: 93% B at 0 min, 93% B at 0.5 min, 40% B at 19 min, 0% B at 28 min and 33 min, returned to 93% B at 36 min, kept at 93% B until 45 min. The column was maintained at 30 °C, the sample temperature was 4 °C, and the injection volume was 1 µL.

The MS/MS instrument was operated in both positive and negative ion modes with the following parameters: resolution 70 k, normalized 3e6 AGC target, maximum injection time 50 ms, mass scan range (*m*/*z*) between 66.7 to 1000. The MS2 data were acquired for each polarity in the top5 program with 0.4 m/z isolation and fragmentation at NCE 15, 30, and 50. For the targets, the data were analyzed with Tracefinder© (version 4.1, Thermo Fisher, Waltham, MA, USA).

For the untargeted analysis, the data were analyzed and curated in Compound Discoverer (CD version 3.3, Thermo Fisher). The candidate identifications were based on MS/MS spectral matching with mzcloud (level 2 identification). The compound intensity rates were similar between the amazake samples, whereas the control rice sample had a lower intensity, indicating that the biomass was lower for this sample. The data were normalized between samples.

### 2.9. Volatile Aroma Compound Analysis (HS-SPME-GC/MS)

The volatile aroma analysis was carried out on amazakes that had been prepared with kojis from *A. oryzae* and *B. amyloliquefaciens* and fermented at 55 °C for 16 h. Steamed white rice was used as a negative control and distilled water as a blank sample. All samples were analyzed in triplicate and kept at −23 °C until their analysis.

The extraction of the samples for the HS-SPME-GC/MS analysis was performed following the protocol outlined by Feng et al. with minor modifications [26].

A solid-phase microextraction (SPME) fiber of polydimethylsiloxane–divinylbenzene (PDMS/DVB; 65 µm) was used for the extraction of the volatile compounds. After the vials containing the samples were incubated at 45 °C for 15 min, the SPME fiber was inserted into the headspace at 45 °C for 40 min and then inserted into the GC injection port. The GC/MS analysis was performed on a Thermo Scientific TRACE 1310 gas chromatograph equipped with a Thermo Scientific Q Exactive Orbitrap mass spectrometry system using an Agilent fused-silica capillary column of cross-linked DB5-MS (30 m × 0.25 mm × 0.25 µm). The GC conditions were as follows: inlet and transfer line temperatures, 250 °C; oven temperature program, 40 °C for 2 min, 5 °C/min to 120 °C for 2 min, 7 °C/min to 220 °C for 5 min, 50 °C/min to 325 °C for 3 min; inlet helium carrier gas flow rate, 1 mL/min; split ratio, 20:1. The electron impact (EI)–MS conditions were as follows: ionization energy, 70 eV; ion source temperature, 250 °C; full scan m/z range, 30—350 Da; resolution, 60,000; AGC target, 1e6; maximum IT, 200 ms. The data were acquired with Thermo TraceFinder 4.1 and analyzed with Thermo Compound Discoverer 3.3 software.

### 2.10. Sensory Analysis: Consumer Study (Check-All-That-Apply and Hedonic Test)

A consumer study was performed to assess acceptance and attribute descriptions for the two amazakes that had been fermented for 16 h at 55 °C and inoculated with either *A. oryzae* or *B. amyloliquefaciens*. A total of 105 consumers participated in the study (51% male and 49% female, ages 20–25). The study was conducted in a tasting room with a controlled temperature (22 ± 2 °C) and non-natural light (fluorescent). Approximately 50 g of each amazake sample was served at room temperature in clear plastic cups with small plastic spoons. For the first part of the consumer study (the hedonic test), the participants tested each sample and rated their liking using a 7-point hedonic scale (1 = extremely dislike, 7 = extremely like) for texture, aroma, taste, sweetness, and visual and overall perception. For the second part of the consumer study, a check-all-that-apply (CATA) test was used for the taste evaluation. The CATA questionnaire contained 20 attributes (which had been selected from a pretasting that included four persons from Harvard’s Science and Cooking Program).

The consumers attended the tasting once. The evaluation order was randomized and the samples were coded with 3-digit random numbers. Amazakes from both *A. oryzae* and *B. amyloliquefaciens* were served at the same time and the consumers were prompted to rinse their mouths with water and unsalted crackers before continuing to the next sample.

The analysis of the hedonic test data was carried out using the parametric test procedure to identify significant differences between the *A. oryzae* and *B. amyloliquefaciens* samples. The statistical analysis was performed using XLSTAT Version 2020.4.1. The data from the CATA study were analyzed using Cochran’s Q test with a pairwise comparison approach based on the McNemar–Bonferroni method to identify significant differences between attributes associated with each sample [27].

### 2.11. DNA Extraction, Sequencing, and Processing

The DNA extraction and sequencing analysis was performed on amazakes made with *A. oryzae* and *B. amyloliquefaciens* kojis fermented at 55 °C for 16 h. The total genomic DNA for each amazake was extracted using a DNeasy PowerLyzer PowerSoil Kit (Qiagen, Inc., San Diego, CA, USA) following the manufacturer’s instructions. The DNA was prepared using a transposase-mediated, Tagmentation-based library prep method (Illumina DNA Kit, San Diego, CA, USA, Quarter Volume) with 10 amplification cycles. The index adapter type was IDT^®^ for Illumina DNA Unique Dual 10 bp Indexes, set D. The samples were shotgun-sequenced on the Illumina NovaSeq 6000 using a single SP flow cell lane with paired-end, 100 bp reads at the Bauer Core Facility at Harvard University.

The program fastp (v 0.23.4) was used with the option “detect_adapter_for_pe–cut_tail” to trim adapters and low-quality reads. Bowtie2 (v 2.5.2) was used to filter out reads belonging to *Bacillus licheniformis* (GCF_000011645.1), *Oryza sativa* japonica group Koshihikari (GCA_000164945.1), and *Oryza sativa* japonica group Nipponbare (GCF_001433935.1). Kaiju (v 1.9.2) was used to taxonomically classify both the filtered and unfiltered reads, and the command kaiju2table was used to generate the abundance table.

### 2.12. Sequencing Statistical Analyses

The statistical analyses were performed in R version 4.2.1 [28]. The species richness was calculated using the vegan package [29] and all samples were rarefied to 8,027,727 reads to account for differences in sequencing depth. An analysis of variance (ANOVA) was performed to test the differences in community richness between the different organism-based cultures. Differences based on time points were evaluated using a mixed effect model, with “time point” as a fixed effect and “pair” (same replicate, different time point) as a random effect using the lme4 package [30]. The Bray–Curtis distances were calculated and a nested permutational multivariate analysis of variance (PERMANOVA) was then conducted to analyze the differences in microbial community composition between cultures and time points. The differences in microbial community composition were visualized using non-metric multidimensional scaling ordination.

## 3. Results and Discussion

### 3.1. Production of an Amazake-like Beverage from Bacillus amyloliquefaciens

**Figure 1:**
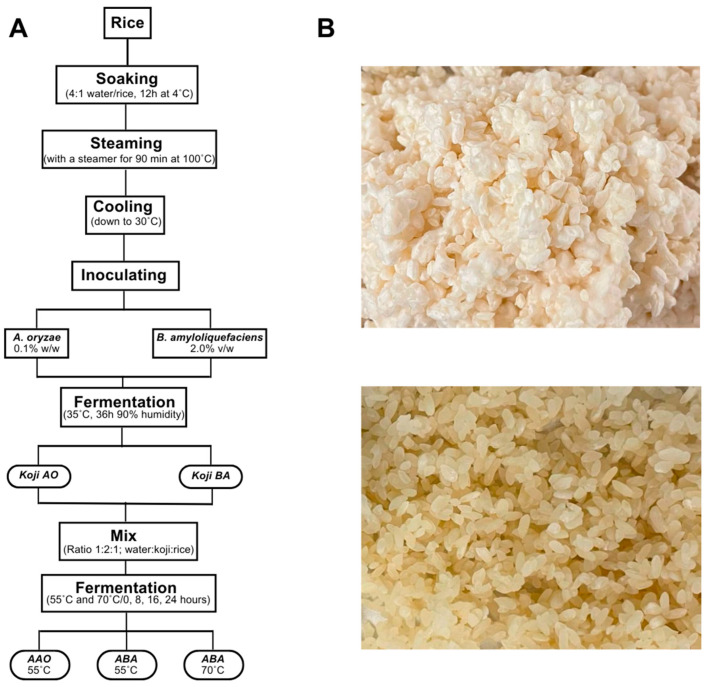
(**A**) Overview of the amazake production process for *A. oryzae* amazake (AAO) and *B. amyloliquefaciens* amazake (ABA) at two different temperatures (55 °C and 70 °C), each fermented for 0, 8, 16, and 24 h. (**B**) Kojis fermented with *A. oryzae* (AO) (**top**) and *B. amyloliquefaciens* (BA) (**bottom**).

A sweet-tasting beverage similar to traditional amazake was successfully created using *B. amyloliquefaciens*. This novel beverage was produced following a process that is equivalent to the traditional method of amazake production, with the exception of using a different microbe, *B. amyloliquefaciens* (NCIMB 12077), instead of *A. oryzae* (W-20) (Figure 1). The preliminary tastings showed that the new beverage shares a key characteristic with traditional amazake; it is sweeter than the raw ingredients, suggesting the breakdown of carbohydrates into di- and monosaccharides. It also had a more complex flavor profile than the original substrate (rice), suggesting the generation of diverse volatile and non-volatile flavor compounds during the fermentation reaction. Interestingly, the flavor profile of the novel amazake seemed distinct from that of traditional amazake, highlighting its unique characteristics. The following sections present the characterization of this novel beverage and compares its properties and production process to those of traditional amazake.

Although our results show that A. oryzae has a higher saccharification rate and enzymatic activity level, these attributes alone do not ensure identical functionality or yield rates when compared to *B. amyloliquefaciens*. The characteristics of these microorganisms are different, and even if present in the same quantity, their performance and outcomes can vary significantly. Therefore, we measured the ability of *B. amyloliquefaciens* to produce metabolites and saccharify the medium to understand its viability in the amazake production process.

### 3.2. Comparison of Reducing Sugar Contents during Amazake Production with Aspergillus oryzae and Bacillus amyloliquefaciens

**Figure 2:**
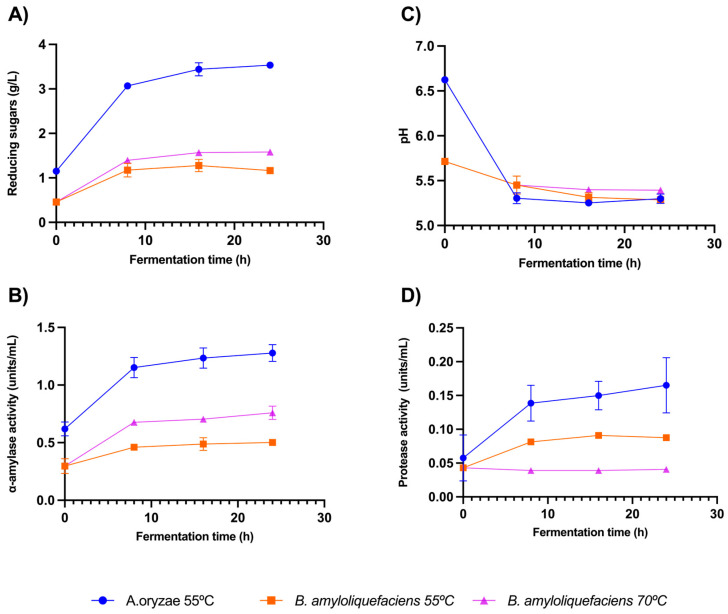
(**A**) Reducing sugar content, (**B**) pH, (**C**) α-amylase activity, and (**D**) protease activity values measured during fermentation for amazake samples produced with *A. oryzae* at 55 °C, *B. amyloliquefaciens* at 55 °C, and *B. amyloliquefaciens* at 70 °C. Three fermentation replicates were performed for each condition. Results are presented as means ± standard deviations.

Saccharification is one of the most distinguishing characteristics of traditional amazake. As the fermentation progresses, enzymes such as amylases and glucosidases that are secreted by *A. oryzae* during the koji fermentation break down carbohydrates to di- and monosaccharides, which can be detected by our taste buds [10]. Saccharification can be estimated by measuring the reducing sugar content (RS). The sugars produced by *A. oryzae* are mostly reducing monosaccharides (xylose, fructose, and glucose), whereas *B. amyloliquefaciens* mainly produces the reducing disaccharide maltose [21,22]. We initially compared the RS contents for amazakes produced at the traditional fermentation temperature of 55 °C for both microorganisms, namely an amazake produced with *A. oryzae* (AAO) and another produced with *B. amyloliquefaciens* (ABA) (Figure 2A). Both microorganisms produced RS at a higher rate during the first 8 h of the fermentation process; this corresponds to the commonly used fermentation time in traditional amazake recipes [4]. After 8 h, the RS contents for both fermentations began to decrease and eventually approached a plateau. The RS content at each time point was significantly different (*t*-test; *p* < 0.001).

We observed that the RS values were higher for the AAO amazake at every time point of the fermentation process (*p* < 0.001), with the highest concentration being found at 24 h (353.0 g/L), as compared to the ABA amazake, which showed the highest concentration at 16 h (127.9 g/L). Ultimately, after 24 h, the saccharification came to an end in both cases, probably due to a substrate or enzyme limitation or as a result of product accumulation and consequent product inhibition [31].

The rate of saccharification from starch hydrolysis is governed both by the enzymatic activity and initial concentration of the starch substrate, the latter of which was equal in both of our amazakes [14]. The higher RS content in AAO indicates that the saccharification efficiency is higher for *A. oryzae.* However, given that sugar metabolism involves many pathways with complex interactions [32], it is challenging to know the exact reasons for the changes in the RS values. Previous studies have found that the cooperation between enzymes such as amylolytic enzymes and reductases allow microorganisms, particularly *A. oryzae*, to effectively metabolize carbohydrates [22]. Further investigations are necessary to determine the specific sugars produced during both saccharification processes. Our findings emphasize *A. oryzae* as the optimal microbial inoculum source for maximal saccharification efficiency under these conditions (i.e., same temperature and ratio of ingredients) (*p* < 0.01). However, it is noteworthy that saccharification with *B. amyloliquefaciens* remains feasible, although with potentially different kinetics.

Unlike *A. oryzae*, *B. amyloliquefaciens* secretes thermal-resistant amylases [14]. Hence, we conducted additional saccharification experiments with ABA at 70 °C, and were able to show a significantly higher RS content compared to the fermentation at 55 °C (*p* < 0.05). Similarly to the 55 °C ABA, the ABA produced at 70 °C exhibited rapid RS production within the initial 8 h, followed by a plateau between 8 and 16 h. These results suggest that higher temperatures enhance the saccharification process in ABA, potentially due to increased amylase activity or reduced bacterial proliferation by *B. amyloliquefaciens* [33]. Further investigations employing varying saccharification temperatures are necessary to validate these findings. Amazake production at higher temperatures is an exciting prospect because it could prevent the proliferation of food-borne pathogens associated with lower temperatures. It opens avenues for secondary fermentations utilizing the fermented sugars produced with *B. amyloliquefaciens*.

### 3.3. Comparison of α-Amylase Activity during AAO and ABA Production

To gain a deeper understanding of saccharification during fermentation, the α-amylase activity was measured for multiple time points. The α-amylases are extracellular enzymes that randomly hydrolyze starch into disaccharides and oligosaccharides, ultimately leading to the production of monosaccharides and sugar alcohols through carbohydrate metabolism [22].

The trends observed for the RS contents mirrored the α-amylase activity rates for all samples (Figure 2B), with all measurements being significantly different for both time points across and between microorganisms. The AAO samples consistently exhibited higher enzymatic activity rates at every time point (*p* < 0.001), reaching a peak at 24 h (306.8 units/mL), while ABA also peaked at 24 h, albeit with a lower value (120.3 units/mL). Notably, at the start of the fermentation (0 h), AAO displayed significantly higher ɑ-amylase activity (148.5 units/mL) compared to ABA (71.2 units/mL), which is in line with previous findings for koji fermentation with the same organisms [22]. During the initial 8 h, the ɑ-amylase activity remained higher in AAO (276.4 units/mL) compared to ABA (110.4 units/mL). This is possibly attributable to different optimal temperature preferences for the enzymes in the two organisms [4]. It could also be due to greater production of extracellular enzymes by *A. oryzae* during koji fermentation compared to *B. amyloliquefaciens*.

As was also done for the RS content, the ɑ-amylase activity for the ABA was measured at both 70 °C and 55 °C (Figure 2B). The ɑ-amylase activity was higher for amazakes prepared at 70 °C than at 55 °C for all data points (*p* < 0.05). This was consistent with the fact that *B. amyloliquefaciens* produces thermoresistant ɑ-amylases, which thrive at 65 °C [14].

### 3.4. Comparison of Protease Activity and pH during AAO and ABA Production

Proteases break down proteins into peptides, which can contribute flavor precursors, mouthfeel, and enhance the α-amylase activity [34]. We measured the protease activity to better understand the dynamics of the fermentation. This aspect had not been explored previously in amazakes prepared with either of the two microbes.

The protease activity followed a similar trend to the α-amylase activity, with overall higher levels in AAO than in ABA (*p* < 0.001). AAO showed a higher activity rate during the first 8 h and reached a maximum activity rate of 0.16 units/mL at 24 h (Figure 2D). In ABA, the maximum activity rate of 0.09 units/mL occurred at 16 and 24 h. This aligns with the literature, indicating that *A. oryzae* produces more proteases than *B. amyloliquefaciens* during koji fermentation [21]. After 10 h, the protease activity in both samples began to plateau, possibly due to the availability of rice protein, enzyme limitations, or the accumulation of inhibitory products. The protease activity changed significantly over time for each microorganism (*p* < 0.001).

The optimal temperatures for protease enzymes of *A. oryzae* and *B. amyloliquefaciens* fall within the ranges of 55 °C–60 °C [35] and 50 °C–60 °C [36], respectively. This could explain why the protease activity for ABA was higher at 55 °C than at 70 °C, i.e., the reverse of what was observed for the α-amylase activity.

The enzymatic activity is governed not only by temperature but also by pH. The amazake inoculated with *A. oryzae* started with a higher pH of 6.6, whereas the amazake inoculated with *B. amyloliquefaciens* had an initial pH of 5.7. Over time, the pH values of all three amazakes landed between 5 and 5.5; both values correspond with the literature for koji fermentation (Figure 2C) [37]. This pH range may be less conducive to the proteases of *B. amyloliquefaciens*, which function optimally above pH 7.0 [38], while *A. oryzae* proteases thrive in the range of pH 4.5 to 6.0 [35].

Overall, our results can be explained by the fact that *B. amyloliquefaciens* proteases are sensitive both to higher temperatures and lower acidity levels. The variations in protease activity highlight the distinct enzymatic behaviors of the two microbial communities during amazake fermentation.

### 3.5. Comparison of the Metabolomic Profiles for AAO and ABA

In addition to the main saccharides, amazake contains over 300 compounds [2]. These include all 20 amino acids derived from rice proteins broken down by *A. oryzae* proteases, as well as various organic acids formed during carbohydrate metabolism. Both amino acids and organic acids influence the acidity of the culture environment and its habitability for microbes, as well as the final flavor and organoleptic characteristics of the amazake. In this study, we used liquid chromatography–mass spectrometry to evaluate differences in the contents of amazake metabolites.

The untargeted and targeted data analyses revealed distinct differences between the metabolite profiles of the two amazakes. Overall, both amazakes showed similar compound intensity rates to each other, and both intensity rates were higher than for the rice control (Supplementary Figure S1). The principal component analysis (PCA) of the untargeted data showed that the two amazakes have clearly different metabolomic profiles from each other (Supplementary Figure S2). PC1 (46.5%) and PC2 (38.2%) accounted for most of the variance, with the main separation occurring between ABA and AAO and the control rice. The replicates for each type of amazake were grouped closely together, which indicates consistent fermentation outcomes.

The targeted data analysis (i.e., compounds that were confirmed with a standard) corroborated these findings, with the PCA showing a clear separation between the amazake samples fermented with different microorganisms. PC1 (66.4%) and PC2 (19.65) revealed distinct clustering of the AAO and ABA samples, with AAO exhibiting a richer profile of specific metabolites compared to ABA (Supplementary Figure S3).

The free amino acids detected in both the AAO and ABA amazakes were nearly identical (Table 1). One exception was for cysteine, which was only produced in the AAO fermentation. Quantitatively, the AAO samples had higher levels of most amino acids except for alanine, asparagine, and histidine, which were present in higher amounts in the ABA samples. These results line up with our previous observations, where AAO demonstrated higher protease activity (Figure 2D).

The amino acids detected in our study matched those commonly found in amazake fermented with *A. oryzae*, as described in the previous literature [4]. However, we also identified additional amino acids not reported in some studies of koji fermentation with *A. oryzae* and *B. amyloliquefaciens* [22]. These differences may arise from variations in the type of rice used or the duration of the fermentation.

For the organic acids, the AAO samples exhibited significantly higher abundance levels of citric, malic, fumaric, and succinic acids compared to ABA (*p* < 0.005) (Table 2). Additionally, both amazakes produced coumaric acid, with the highest abundance level found in AAO, while shikimic acid was only detected in ABA samples [20]

Some reports indicate the presence of citric acid, succinic acid, malic acid, and fumaric acid in traditional amazake [10,39], which is consistent with our findings. Additionally, pyruvic acid has previously been found, which was not detected in our study. Coumaric acid and shikimic acid were detected for the first time here, with shikimic acid being newly generated in ABA. Shikimic acid has previously been detected in koji fermentation with *B. amyloliquefaciens* [22].

Overall, AAO fermentation results in a richer metabolite profile than ABA, showcasing higher concentrations of both amino acids and organic acids. These differences reflect the distinct fermentation pathways utilized by *A. oryzae* and *B. amyloliquefaciens*.

### 3.6. Sensory Analysis

**Figure 3:**
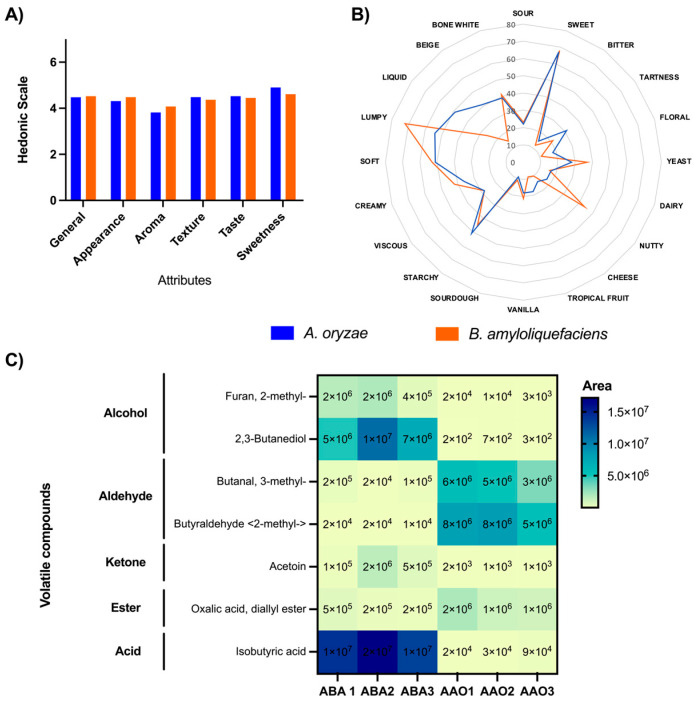
Sensory analysis and volatile aroma compounds in *A. oryzae* and *B. amyloliquefaciens* amazakes fermented at 55 °C for 16 h. (**A**) Hedonic test (1 = extremely dislike to 9 = extremely like) and (**B**) check-all-that-apply test. There were no statistically significant differences in any of the attributes. Results are reported as means; *n* = 105. (**C**) Volatile aroma compounds detected. Three fermentation replicates were performed for each condition. Results are reported in area.

We hypothesized that the distinct metabolomic profiles of the two amazakes would be reflected in the sensory attributes of the amazakes. The preliminary tastings indicated that this was indeed the case. To explore this further, we performed a consumer study that assessed acceptance and sensory attributes for the novel ABA amazake in comparison to the traditional AAO amazake. To assess their acceptance, a hedonic test was conducted that measured ‘liking’ in terms of aroma, texture, appearance, sweetness, taste, and the overall profile. The scores for all of the attributes fell between 3.8 and 4.9 out of 9, with no significant differences between samples, suggesting that the study participants found the flavors to be similar. In particular, the scores for ‘overall liking’ for both amazakes fell between “neither like nor dislike” and “dislike a little” (mean values of 4.52 and 4.47 for AAO and ABA, respectively), with no significant differences detected between samples, suggesting that the study participants found both amazakes about equally palatable (Figure 3A).

The consumer study also assessed the sensory attributes with a CATA questionnaire (Figure 3B). The consumers described both amazakes as sweet, with AAO being perceived as more fruity, tart, and liquid, whereas the ABA was described as more nutty, yeasty, and lumpy (Figure 3). However, none of these attributes were deemed significantly different between AAO and ABA.

The flavor profile of an amazake relates to the amino acid and organic acid contents. Arginine, which we found to be more abundant in AAO, has been reported to produce pleasant, fruity, and sour aromas at pH 5.2. Similarly, proline, which is also more abundant in AAO, produces pleasant, flowery, and fragrant aromas [40]. In addition, fumaric acid, which is also more abundant in AAO, tastes fruity [41]. Taken together, these findings correspond with the sensory data, which describe AAO as being more tart and fruity than ABA.

In summary, although the metabolic profile analysis showed that the amazakes are different, this was not reflected in the sensory analysis data, indicating that these differences are not noticeable to the average consumer.

### 3.7. Comparison of Volatile Aroma Compounds during AAO and ABA Production

Gas chromatography–mass spectrometry was performed to further understand the molecular basis of our sensory results. Flavor is composed of taste and aroma, with volatile aroma compounds contributing to the aroma, thereby ultimately contributing to the flavor. The experiment identified a total of 9 volatile compounds that were produced during the fermentation from rice to amazake (Figure 3C). These included 2 alcohols (2-methyl-furan and 2,3-butanediol), 2 aldehydes (3-methyl-butanal and 2-methyl-butyraldehyde), 1 ketone (acetoin), 1 carboxylic acid (isobutyric acid), and 1 carboxylic ester (diallyl ester oxalic acid).

The most abundant compounds in the AAO fermentation compared to ABA were 2-methyl-butyraldehyde (which has a cocoa, musty, green, and malty aroma), 3-methyl-butanal (chocolaty, peachy aroma), and diallyl ester oxalic acid (floral aroma). The greater abundance of these specific compounds aligns with earlier research [42], which showed that during koji fermentation on wheat and soybean, *A. oryzae* produces a greater abundance of aldehyde compounds compared to *B. amyloliquefaciens*.

Conversely, the most abundant compounds in the ABA fermentation compared with AAO were isobutyric acid (which has a sour, cheesy, dairy-like, buttery, and rancid aroma and contributes to the characteristic smell of many dairy products), 2,3-butanediol (fruity, creamy, buttery; used as a flavoring agent, common in other fermented foods and beverages), and acetoin (sweet, buttery, creamy, and dairy-like, used as a flavor enhancer in butter, cream, and dairy-flavored products). The greater abundance of these specific ketones and alcohols in the ABA fermentation aligns with earlier research [42], which demonstrated that during koji fermentation on wheat and soybean, *B. amyloliquefaciens* exhibits a greater abundance of these compounds. However, our study diverged by detecting isobutyric acid, a compound not found in the earlier research.

Overall, the volatile aroma profile helps explain our sensory results from the CATA test, which described the AAO to be tarter and fruitier and ABA to have more nutty and yeast-like aromas. It should be noted, however, that aroma is a complex concept determined by combinations of many compounds. Additionally, this experiment measured volatiles that developed during the fermentation only, and did not include volatiles that were already present in the rice or koji at the start of the fermentation at 0 h. This could help explain any other aromas detected by the sensory panel, such as the nutty flavors associated with ABA.

### 3.8. Microbial Community of Amazake Cultures Based on AAO and ABA Production

Fermented products rely on the specific microbial communities present in them, which can influence the flavor, nutritional value, and safety of the final product [43]. Our study investigated the initial and final microbial communities to uncover any shifts in species dominance and diversity, as well as potential implications for food quality and safety. This has not previously been done for amazakes produced with either of the two microbes.

**Figure 4:**
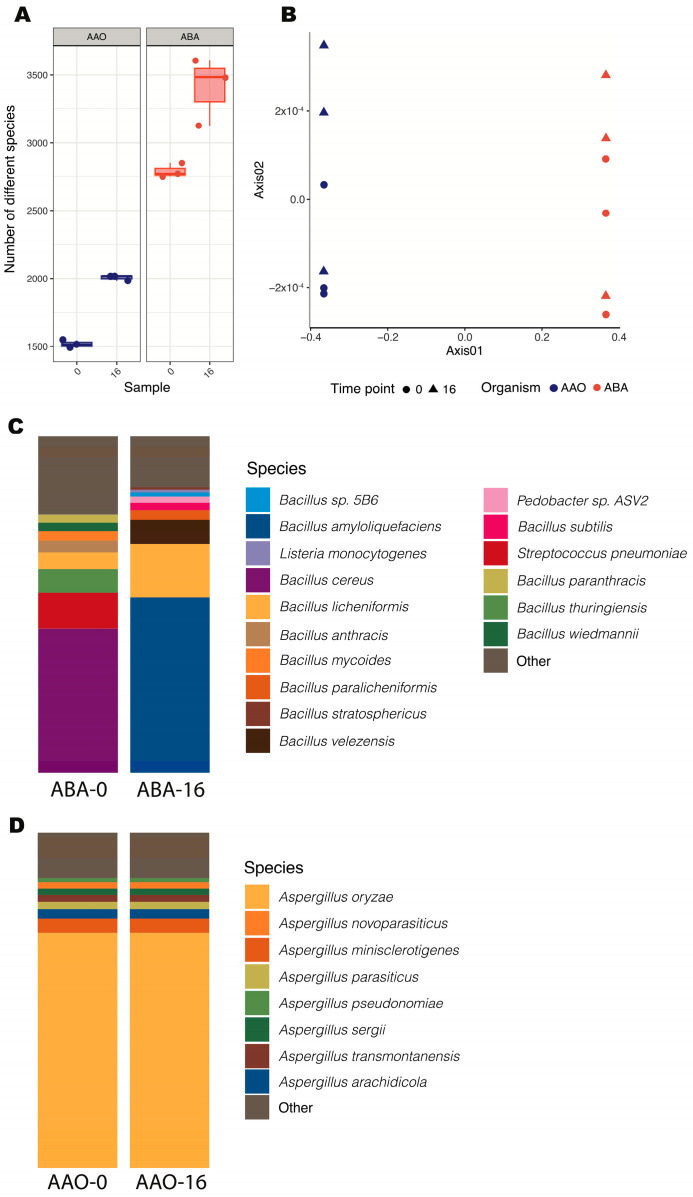
Microbial communities of *A. oryzae* and *B. amyloliquefaciens* amazakes: (**A**) numbers of different species in the microbial communities of ABA and AAO amazakes at 0 and 16 h; (**B**) Bray–Curtis dissimilarity rates between compositions of microbial communities in ABA and AAO amazakes at 0 and 16 h; (**C**) taxonomic characterization of the dominant microorganisms in the microbial communities of ABA amazake and (**D**) AAO amazake at 0 and 16 h. All amazakes were fermented at 55 °C.

First, we compared the microbial community structure of each amazake at time 0 and found that the initial number of microbial species (richness) in ABA was nearly double that of AAO (Figure 4A; F_1,10_ = 53.15, *p*-value < 0.001). Simultaneously, the microbial community compositions differed significantly between AAO and ABA (Figure 4B; R^2^ = 0.876, *p*-value = 0.001). These results show that the primary organism in an amazake culture greatly determines the whole microbial community, thereby determining the physicochemical, gastronomical, and nutritional properties of the final product.

Secondly, we investigated the changes in microbial community structure between the initial and final time points of each amazake. After 16 h of fermentation, the microbial richness was greater than at time 0 for both AAO (Figure 4A; X^2^ (1, N = 6) = 428.41, *p*-value < 0.001) and ABA (Figure 4A; X^2^ (1, N = 6) = 17.72, *p*-value < 0.001). Likewise, the microbial community composition was influenced, although to a lesser extent, by the time point (Figure 4B; R^2^ = 0.01516, *p*-value = 0.290). Interestingly, we noted that some of the 16 h amazakes were still very similar in community composition to the initial amazakes (0 h) for both microorganisms (Figure 4A), potentially signaling some variability in the rates of compositional change.

Specifically, the *B. amyloliquefaciens* amazake demonstrated a shift towards predominance by the inoculated bacteria from 0 to 16 h, with *B. amyloliquefaciens* increasing in abundance over time to become the dominant species. This is interesting because it shows that *B. amyloliquefaciens* did not establish dominance during the koji fermentation but only managed to do this during the amazake process. The top species present at time 0 h were *Bacillus cereus* and *Streptococcus pneumoniae*, which are commonly associated with food poisoning and human diseases [44,45]. By the end of the fermentation (16 h), these were replaced by microorganisms such as *Bacillus licheniformis*, a common probiotic that can be found in a variety of foods [46], as well as *Bacillus velezensis* (Figure 4C). The latter shares a close phylogenomic resemblance with *B. amyloliquefaciens* and is commonly found in fermented food products; it is characterized by its strong proteolytic activity during fermentation processes [47]. Certain strains of *B. amyloliquefaciens* have been reported to inhibit the synthesis of toxic substances, including toxic compounds produced by *Bacillus cereus* in the food industry [12], thereby highlighting the potential for producing fermented products inoculated with these beneficial bacteria. In contrast, fungi maintain dominance throughout the fermentation period of AAO amazakes, indicating stability in the microbial community. In addition to the dominant species *Aspergillus oryzae*, many other *Aspergillus species* were detected, including *Aspergillus minisclerotigenes* and *Aspergillus arachidicola* (Figure 4D). These two species have previously been associated with the production of aflatoxins [48]. It is known that a sequencing analysis may not be sufficient to accurately identify different species within the *Aspergillus flavus* group, which encompasses *Aspergillus oryzae*, *Aspergillus flavus*, *Aspergillus minisclerotigenes*, and *Aspergillus arachidicola*, among others [49]. Therefore, we hypothesize that these species may be misidentified and could all potentially belong to *Aspergillus oryzae*; further analyses are required to confirm this hypothesis.

## 4. Conclusions

In summary, our study successfully produced a sweet amazake beverage using *Bacillus amyloliquefaciens* (NCIMB 12077) (ABA). A consumer panel indicated similar acceptability comparable to traditional amazake, with flavors of nuts and yeast. The underlying metabolomic and metagenomic analyses revealed distinct profiles between the ABA and AAO amazakes. The amylase activity was higher for the ABA amazake produced at 70 °C, whereas the protease activity was higher when it was produced at 55 °C. This research offers promising insights into the potential of *B. amyloliquefaciens* as the primary microorganism for fermenting beverages like amazake, representing a significant stride forward in the realm of fermented low-alcohol beverage production. Moreover, the prospect of fermentation at elevated temperatures opens avenues for further exploration and application in diverse fermented food products.

## Figures and Tables

**Table 1:** Free amino acid compositions in *A. oryzae* and *B. amyloliquefaciens* amazakes after 16 h of fermentation at 55 °C.

Amino Acids	AAO	ABA	*p*-Value
Alanine	4 × 10^9^	7.27 × 10^9^	3.55 × 10^−2^
Arginine	3 × 10^11^	1.29 × 10^11^	1.56 × 10^−6^
Asparagine	7 × 10^8^	1.57 × 10^9^	4.32 × 10^−2^
Aspartate	1 × 10^10^	1.12 × 10^9^	6.30 × 10^−5^
Cysteine	2 × 10^7^	N/F	^−^
Glutamate	2 × 10^10^	4.42 × 10^9^	7.39 × 10^−4^
Glutamine	1 × 10^10^	1.36 × 10^10^	8.92 × 10^−2^
Histidine	1 × 10^10^	1.64 × 10^10^	1.88 × 10^−4^
Isoleucine	2 × 10^11^	1.40 × 10^11^	7.86 × 10^−3^
Leucine	1 × 10^11^	6.79 × 10^10^	1.11 × 10^−1^
Lysine	4 × 10^10^	1.64 × 10^10^	5.72 × 10^−6^
Methionine	4 × 10^10^	2.88 × 10^10^	6.87 × 10^−2^
Phenylalanine	2 × 10^11^	8.36 × 10^10^	7.22 × 10^−3^
Proline	2 × 10^11^	2.92 × 10^10^	2.02 × 10^−5^
Serine	3 × 10^9^	1.70 × 10^9^	2.86 × 10^−2^
Threonine	6 × 10^9^	5.76 × 10^9^	9.17 × 10^−1^
Tryptophan	3 × 10^10^	9.64 × 10^9^	5.52 × 10^−5^
Tyrosine	4 × 10^10^	2.14 × 10^10^	2.05 × 10^−3^
Valine	2 × 10^11^	9.37 × 10^10^	4.00 × 10^−3^
GABA	1 × 10^10^	4 × 10^8^	0.00808

The values are the means of triplicate experiments; *p* < 0.01. N/F means not found. Results are reported by area.

**Table 2:** Organic acid compositions in *A. oryzae* and *B. amyloliquefaciens* amazakes after 16 h of fermentation at 55 °C.

Organic Acids	AAO	ABA	*p*-Value
Citrate	8 × 10^10^	2 × 10^9^	0.00067
Coumaric acid p or o	4 × 10^9^	2 × 10^8^	0.00382
Fumarate	1 × 10^9^	5 × 10^7^	1 × 10^−5^
Malate	1 × 10^10^	1 × 10^9^	0.00054
Shikimic acid	N/F	2 × 10^7^	-
Succinate	1.33 × 10^9^	9.96 × 10^8^	0.00283

The values are the means of triplicate experiments; *p* < 0.01. N/F means not found. Results are reported by area.

## Data Availability

The dataset and code generated during the current study are available in the github repository, accessed in 1 April 2024 (https://github.com/AlexTouceda/amazake_comparison).

## Supplementary Materials

The following supporting information can be downloaded at https://www.mdpi.com/article/10.3390/foods13132012/s1: Figure S1. Panel of the untargeted data, which shows how by using Compound Discoverer, a median centering process was performed. Figure S2. Untargeted overview of the LC-MS/MS dataset—PCA of amazake samples made with varying inoculum permutations at 55 °C for 16 h. Figure S3. Liquid chromatography–mass spectroscopy (LC-MS/MS) datasets of primary metabolites for the amazake samples produced at 55 °C for 16 h.

## References

[1] Ito K., Matsuyama A. (2021). Koji molds for japanese soy sauce brewing: Characteristics and key enzymes. J. Fungi.

[2] Kurahashi A. (2021). Ingredients, functionality, and safety of the japanese traditional sweet drink amazake. J. Fungi.

[3] Saigusa N., Ohba R. (2007). Effects of koji Production and Saccharification Time on the Antioxidant Activity of amazake. Food Sci. Technol. Res..

[4] Oguro Y., Nakamura A., Kurahashi A. (2019). Effect of temperature on saccharification and oligosaccharide production efficiency in koji amazake. J. Biosci. Bioeng..

[5] Kageyama S., Inoue R., Hosomi K., Park J., Yumioka H., Suka T., Kurohashi Y., Teramoto K., Syauki A.Y., Doi M. (2021). Effects of malted rice amazake on constipation symptoms and gut microbiota in children and adults with severe motor and intellectual disabilities: A pilot study. Nutrients.

[6] Maruki-Uchida H., Sai M., Yano S., Morita M., Maeda K. (2020). Amazake made from sake cake and rice koji suppresses sebum content in differentiated hamster sebocytes and improves skin properties in humans. Biosci. Biotechnol. Biochem..

[7] Nagao Y., Takahashi H., Kawaguchi A., Kitagaki H. (2021). Effect of Fermented Rice Drink “Amazake” on Patients with Nonalcoholic Fatty Liver Disease and Periodontal Disease: A Pilot Study. Reports.

[8] Wang Y., Wu J., Lv M., Shao Z., Hungwe M., Wang J., Bai X., Xie J., Wang Y., Geng W. (2021). Metabolism Characteristics of Lactic Acid Bacteria and the Expanding Applications in Food Industry. Front. Bioeng. Biotechnol..

[9] Oda Y., Ichinose Y., Yamauchi H. (2002). Utilization of *Lactobacillus amylovorus* as an Alternative Microorganism for Saccharifying Boiled Rice. Food Sci. Technol. Res..

[10] Oguro Y., Nishiwaki T., Shinada R., Kobayashi K., Kurahashi A. (2017). Metabolite profile of koji amazake and its lactic acid fermentation product by *Lactobacillus sakei* UONUMA. J. Biosci. Bioeng..

[11] Woldemariam Yohannes K., Wan Z., Yu Q., Li H., Wei X., Liu Y., Wang J., Sun B. (2020). Prebiotic, Probiotic, Antimicrobial, and Functional Food Applications of *Bacillus amyloliquefaciens*. J. Agric. Food Chem..

[12] Eom J.S., Choi H.S. (2015). Inhibition of bacillus cereus growth and toxin production by *Bacillus amyloliquefaciens* RD7-7 in fermented soybean products. J. Microbiol. Biotechnol..

[13] Wang Q., Wang F., Zhou Y., Li X., Xu S., Jin Q., Li W. (2023). *Bacillus amyloliquefaciens* SC06 Relieving Intestinal Inflammation by Modulating Intestinal Stem Cells Proliferation and Differentiation via AhR/STAT3 Pathway in LPS-Challenged Piglets. J. Agric. Food Chem..

[14] Syu M.-J., Chen Y.-H. (1997). A study on the α-amylase fermentation performed by *Bacillus amyloliquefaciens*. Chem. Eng. J..

[15] Skowron K., Budzyńska A., Grudlewska-Buda K., Wiktorczyk-Kapischke N., Andrzejewska M., Wałecka-Zacharska E., Gospodarek-Komkowska E. (2022). Two Faces of Fermented Foods—The Benefits and Threats of Its Consumption. Front. Microbiol..

[16] Adams M., Mitchell R. (2002). Fermentation and Pathogen Control: A Risk Assessment Approach. Int. J. Food Microbiol..

[17] Allwood J.G., Wakeling L.T., Bean D.C. (2021). Fermentation and the microbial community of Japanese koji and miso: A review. J. Food Sci..

[18] Alejandra T.-S., Sörensen P.M., Arboleya J.-C. (2022). Comparative Study of Microbial Species Performance in Amazake Production.

[19] Rekdal V.M., Rodriguez-Valeron N., Garcia M.O., Vásquez D.P., Sörensen P.M., Munk R., Keasling J.D. (2023). From lab to table: Expanding gastronomic possibilities with fermentation using the edible fungus Neurospora intermedia. Int. J. Gastron. Food Sci..

[20] Gangadharan D., Sivaramakrishnan S., Nampoothiri K.M., Sukumaran R.K., Pandey A. (2008). Response surface methodology for the optimization of alpha amylase production by *Bacillus amyloliquefaciens*. Bioresour. Technol..

[21] Gil H.J., Lee S., Singh D., Lee C. (2018). Varying inocula permutations (*Aspergillus oryzae* and *Bacillus amyloliquefaciens*) affect enzyme activities and metabolite levels in *Koji*. J. Microbiol. Biotechnol..

[22] Lee D.E., Lee S., Jang E.S., Shin H.W., Moon B.S., Lee C.H. (2016). Metabolomic profiles of *Aspergillus oryzae* and *Bacillus amyloliquefaciens* during rice *Koji* fermentation. Molecules.

[23] Kusumoto K.-I., Yamagata Y., Tazawa R., Kitagawa M., Kato T., Isobe K., Kashiwagi Y. (2021). Japanese traditional *Miso* and *Koji* making. J. Fungi.

[24] Montañez L.J.B. (2020). Cuantificación de azúcares reductores del sustrato en residuos de piña con el método del ácido 3,5-dinitrosalicílico. Quest. Investig. Específica.

[25] Bechman A., Phillips R.D., Chen J. (2012). Changes in Selected Physical Property and Enzyme Activity of Rice and Barley Koji during Fermentation and Storage. J. Food Sci..

[26] Feng Y., Cui C., Zhao H., Gao X., Zhao M., Sun W. (2013). Effect of *koji* fermentation on generation of volatile compounds in soy sauce production. Int. J. Food Sci. Technol..

[27] Valerón N.R., Vásquez D.P., Munk R. (2021). The Pinaceae species, flavor attributes for new culinary spices. Int. J. Gastron. Food Sci..

[28] R Development Core Team (2015). R: A Language and Environment for Statistical Computing.

[29] Oksanen J., Blanchet F.G., Friendly M., Kindt R., Legendre P., McGlinn D., Minchin P.R., O’Hara R.B., Simpson G.L., Solymos P. (2012). *Vegan: Community Ecology Package*; R Package Version 2.0-2. https://github.com/vegandevs/vegan.

[30] Bates D., Mächler M., Bolker B., Walker S. (2015). Fitting Linear Mixed-Effects Models Using lme4. J. Stat. Softw..

[31] Ghosh B., Ray R. (2010). Saccharification of Raw Native Starches by Extracellular Isoamylase of *Rhizopus oryzae*. Biotechnology.

[32] Nakrani M.N., Wineland R.H., Anjum F. (2020). Physiology, Glucose Metabolism.

[33] Abd-Elhalem B.T., El-Sawy M., Gamal R.F., Abou-Taleb K.A. (2015). Production of amylases from *Bacillus amyloliquefaciens* under submerged fermentation using some agro-industrial by-products. Ann. Agric. Sci..

[34] Santos M.V., Banfi S., Santos R., Mota M., Raymundo A., Prista C. (2023). Improving chestnut physicochemical properties through fermentation—Development of chestnut Amazake. Food Chem. X.

[35] de Castro R.J.S., Sato H.H. (2014). Production and biochemical characterization of protease from *Aspergillus oryzae*: An evaluation of the physical–chemical parameters using agroindustrial wastes as supports. Biocatal. Agric. Biotechnol..

[36] Guleria S., Walia A., Chauhan A., Shirkot C.K. (2016). Purification and characterization of detergent stable alkaline protease from *Bacillus amyloliquefaciens* SP1 isolated from apple rhizosphere. J. Basic Microbiol..

[37] Chancharoonpong C., Hsieh P.-C., Sheu S.-C. (2012). Enzyme Production and Growth of *Aspergillus oryzae* S. on Soybean Koji Fermentation. APCBEE Procedia.

[38] Hui C., Wei R., Jiang H., Zhao Y., Xu L. (2019). Characterization of the ammonification, the relevant protease production and activity in a high-efficiency ammonifier *Bacillus amyloliquefaciens* DT. Int. Biodeterior. Biodegrad..

[39] Tukiyama R., Maeda T., Miyashita K., Shikata H., Ishigami Y. (1977). Components in Amazake (Part2) Amino acids, Organic acids Composition, and Other Composition. Jpn. Soy Sauce Res. Inst..

[40] Wong K.H., Aziz S.A., Mohamed S. (2008). Sensory aroma from Maillard reaction of individual and combinations of amino acids with glucose in acidic conditions. Int. J. Food Sci. Technol..

[41] Goldberg I., Rokem J.S. (2009). Organic and Fatty Acid Production, Microbial Defining Statement Introduction Organic Acids Fatty Acids Conclusions Further Reading. Encyclopedia of Microbiology.

[42] Seo H.S., Lee S., Singh D., Park M.K., Kim Y.-S., Shin H.W., Cho S.A., Lee C.H. (2018). Evaluating the headspace volatolome, primary metabolites, and aroma characteristics of *Koji* fermented with *Bacillus amyloliquefaciens* and *Aspergillus oryzaes*. J. Microbiol. Biotechnol..

[43] Campbell-Platt G. (1994). Fermented foods-a world. Food Res. Int..

[44] Jessberger N., Dietrich R., Granum P.E., Märtlbauer E. (2020). The *Bacillus cereus* Food Infection as Multifactorial Process. Toxins.

[45] Park S.-J., Seo K.-H., Han S.-I. (2011). Toxicity study of *Streptococcus pneumoniae* vaccine administrated subcutaneously in rats. Toxicol. Res..

[46] Dindhoria K., Kumar S., Baliyan N., Raphel S., Halami P.M., Kumar R. (2022). Bacillus licheniformis MCC 2514 genome sequencing and functional annotation for providing genetic evidence for probiotic gut adhesion properties and its applicability as a bio-preservative agent. Gene.

[47] Alenezi F.N., Ben Slama H., Bouket A.C., Cherif-Silini H., Silini A., Luptakova L., Nowakowska J.A., Oszako T., Belbahri L. (2021). *Bacillus velezensis*: A treasure house of bioactive compounds of medicinal, biocontrol and environmental importance. Forests.

[48] Pildain M.B., Frisvad J.C., Vaamonde G., Cabral D., Varga J., Samson R.A. (2008). Two novel aflatoxin-producing *Aspergillus* species from Argentinean peanuts. Int. J. Syst. Evol. Microbiol..

[49] Godet M., Munaut F. (2010). Molecular strategy for identification in *Aspergillus* section Flavi: Research letter. FEMS Microbiol. Lett..

