# Supplementary material for: Harnessing Bacillus amyloliquefaciens for Amazake Production: Comparison with Aspergillus oryzae Amazake for Metabolomic Characteristics, Microbial Diversity, and Sensory Profile
Source: Foods. 2024 Jun 26;13(13):2012. doi: 10.3390/foods13132012 (PMC11241664; doi:10.3390/foods13132012)
Supplement: Supplementary file 1 [file foods-13-02012-s001.zip › foods-3015120-supplementary.pdf]

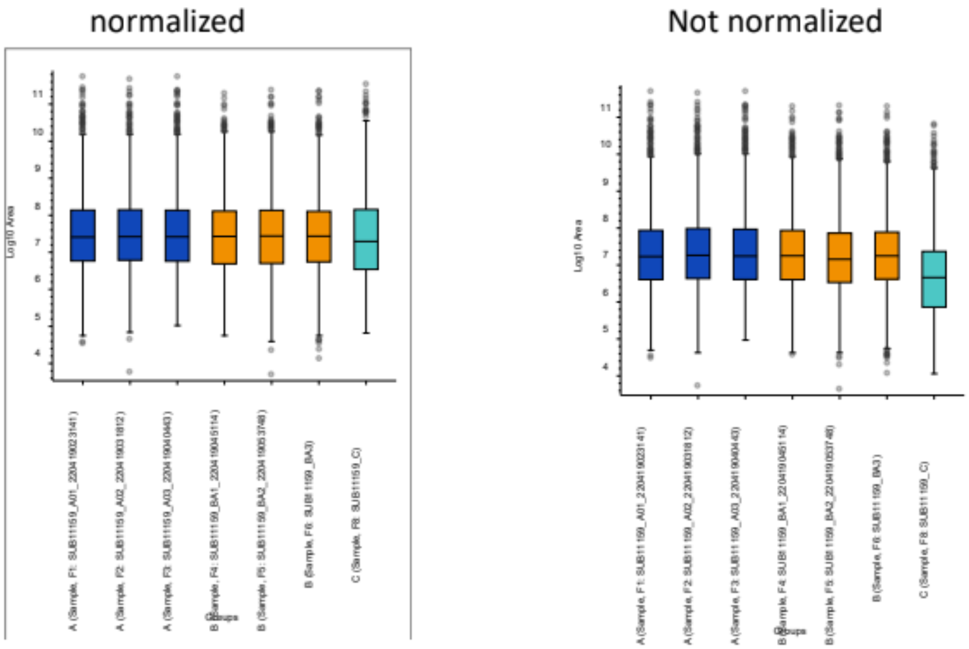

Figure S1. Panel of the untargeted data, where shows how by using Compound Discoverer a median centering was perform.

(A - A. oryzae dark blue, *; B - B. amyloliquefaciens orange, ; C- Control rice, light blue)*

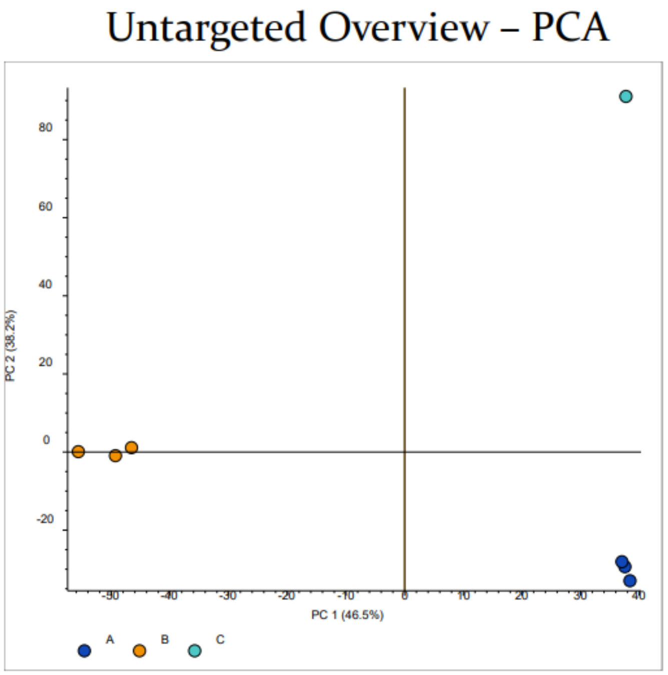

Figure S2. Untargeted overview of the LC-MS/MS dataset – PCA of amazake samples made with varying inocula permutation at 55°C for 16h.

(A - A. oryzae dark blue, *; B - B. amyloliquefaciens orange, ; C- Control rice, light blue)*

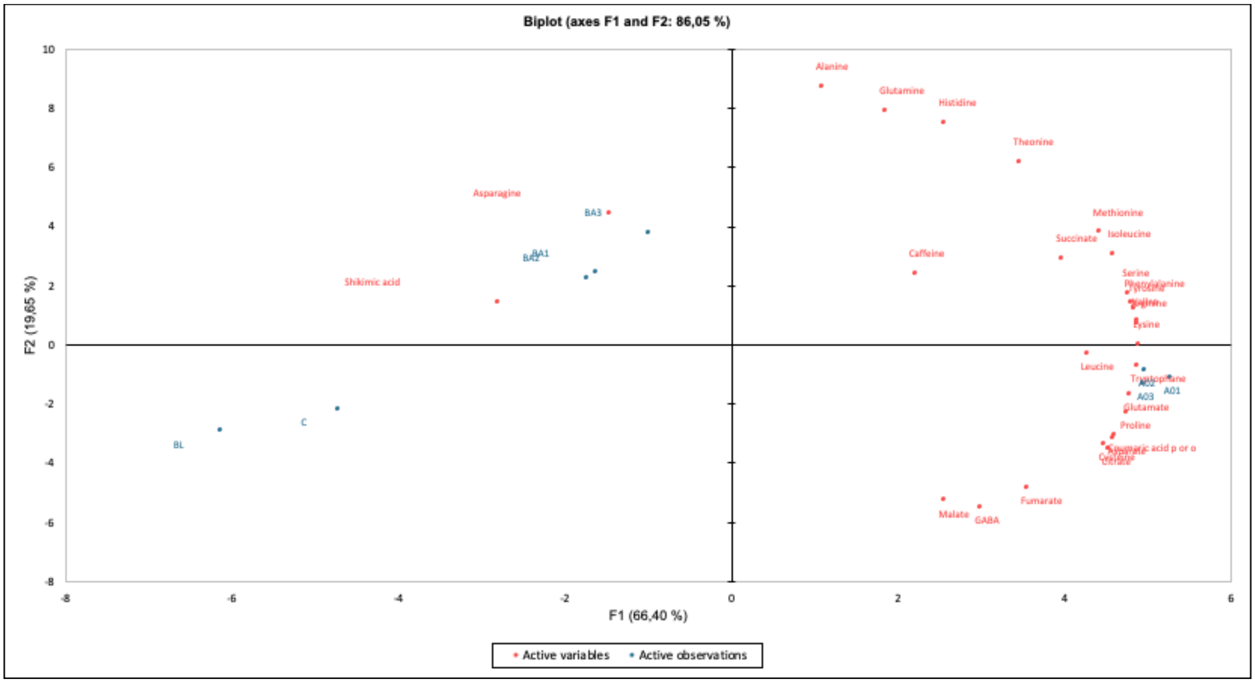

Figure S3. Liquid chromatography- mass spectroscopy (LC-MS/MS) datasets of primary metabolites for the amazake samples at 55°C for 16h.

(The data for targeted compounds are indicated with its names in red; BA1, BA2, BA3- B. amyloliquefaciens, repetitions; AO1, AO2, AO3- A. oryzae, repetitions; BL- Blank; C- Control)
